# Recent Advances in Pain Management: Relevant Protein Kinases and Their Inhibitors

**DOI:** 10.3390/molecules26092696

**Published:** 2021-05-04

**Authors:** Francis Giraud, Elisabeth Pereira, Fabrice Anizon, Pascale Moreau

**Affiliations:** Université Clermont Auvergne, CNRS, Clermont Auvergne INP, ICCF, F-63000 Clermont-Ferrand, France; francis.giraud@uca.fr (F.G.); elisabeth.pereira@uca.fr (E.P.)

**Keywords:** protein kinases, inhibitors, pain

## Abstract

The purpose of this review is to underline the protein kinases that have been established, either in fundamental approach or clinical trials, as potential biological targets in pain management. Protein kinases are presented according to their group in the human kinome: TK (Trk, RET, EGFR, JAK, VEGFR, SFK, BCR–Abl), CMGC (p38 MAPK, MEK, ERK, JNK, ASK1, CDK, CLK2, DYRK1A, GSK3, CK2), AGC (PKA, PKB, PKC, PKMζ, PKG, ROCK), CAMK, CK1 and atypical/other protein kinases (IKK, mTOR). Examples of small molecule inhibitors of these biological targets, demonstrating an analgesic effect, are described. Altogether, this review demonstrates the fundamental role that protein kinase inhibitors could play in the development of new pain treatments.

## 1. Introduction

Pain management is a major public health issue. Indeed, chronic pain affects more than 20% of Europeans, 50% of the elderly population, and costs several hundred billion each year in medical treatment and loss of productivity [1], with an expected increase due to population aging. The situation is also worsened, particularly in North America, by the opioid crisis due to tolerance and physical dependence issues in case of chronic pain treatment with an overdose of opioids [2]. Despite intensive research toward the study of pain mechanisms and significant investments in research and development, nowadays, the majority of analgesics available are based on old drugs or drug classes known for other therapeutic indications, such as anticonvulsants and antidepressants [3,4,5]. This demonstrates the need for the validation of new biological targets in order to better manage pain symptoms. Protein kinases are transferases that catalyze the transfer of a phosphate group from ATP to a hydroxy group of serine/threonine or tyrosine residues of target proteins, leading to the modulation of their activity. Therefore, protein kinases are involved in various physiological processes and signaling pathways and constitute attractive biological targets for the development of new drugs in multiple therapeutic areas. More than 500 human protein kinases have been identified so far, constituting the human kinome. Conventional protein kinases are divided into eight groups based on sequence and function homology. Most of the reported protein kinase inhibitors are ATP-competitive and were developed for their application in cancer therapy (e.g., imatinib, the first tyrosine kinase inhibitor to reach the market). Nowadays, the development of protein kinase inhibitors for other therapeutic applications (e.g., pain, neurodegenerative disease) is emerging [6]. As reviewed by Funez et al. in 2012, among potential targets involved in the physiopathology of pain, it is now well established that various protein kinases, such as PKA (protein kinase A), PKC (protein kinase C), p38 MAPK (p38 mitogen-activated protein kinase), ERK (extracellular signal-regulated kinase) or PKG (protein kinase G), are of high interest for pain management [7]. In addition, a recent review showed that protein kinases such as mTOR (mammalian target of rapamycin) and MNKs (MAPK-interacting kinases) could be targeted in order to manipulate translation regulation signaling for chronic pain treatment [8].

By the means of selected examples, this review aims to highlight the potential application of protein kinase inhibitors in pain. Thus, it does not constitute a presentation of the biology of protein kinases involved in pain and the pharmacology of their inhibitors or an analysis of the current clinical trials in the field. Instead, we review protein kinases that can be targeted by small molecules in the context of pain management. For each protein kinase, inhibitors evaluated for their pain-relieving effect are presented. This account focuses on articles/reviews published since 2010, excluding patents. In the subsequent sections, inhibitors are presented according to the targeted protein kinases.

## 2. Tyrosine Kinase (TK) Group

### 2.1. Tropomyosin-Related Kinases (Trks)

Tropomyosin-related kinases (Trks) are frequently cited as targets for pain application. The tropomyosin-related kinases (Trks) are cell surface receptor tyrosine kinases divided into three homologous isoforms: TrkA, TrkB and TrkC. TrkA was initially identified as an oncoprotein, leading to numerous works dealing with Trk inhibitors with anticancer application. However, the involvement of nerve growth factor (NGF) and its receptor TrkA in chronic pain management is now well established [9]. Therapies that target NGF–TrkA signaling demonstrated significant analgesic activity, showing that small molecule Trk inhibitors would be relevant as new therapeutic approaches to manage several types of chronic pain. Other growth factors of the neurotrophin family (BDNF and NT4 interacting with TrkB; NT3 interacting with TrkC) are also involved in chronic pain. For example, Wang et al., using the cyclic peptide cyclotraxin B, a noncompetitive highly potent and selective TrkB inhibitor (IC_50_ TrkB = 0.3 nM) [10], demonstrated the implication of BDNF/TrkB in the pain signaling pathway [11].

**Figure 1:**
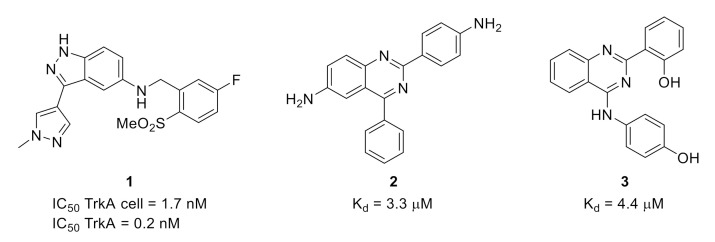
Structures and activities of small molecule Trk inhibitors **1**, **2** and **3**.

The availability of several structures of TrkA in the protein data bank enabled the use of chemoinformatic approaches for the development of new Trk inhibitors. For example, a structure-based drug design approach led to the discovery of 3-pyrazolyl-indazole **1** (Figure 1) as a potent pan-Trk inhibitor with low brain penetration ability and in vivo activity in a thermal hypersensitivity inflammatory pain model induced by Complete Freund’s Adjuvant (CFA) [12].

A structure-based virtual screening with homology-modeled protein structure has been used to identify new TrkA inhibitors such as quinazolines **2** and **3** (Figure 1) [13]. 

**Figure 2:**
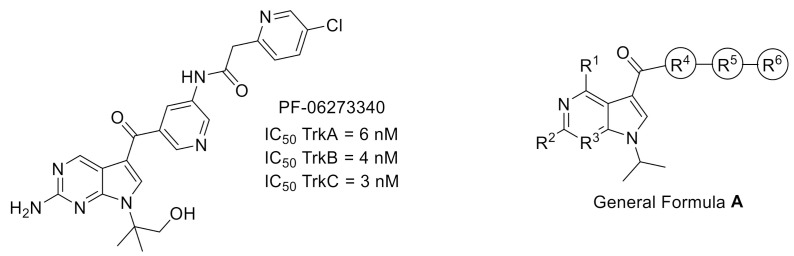
Structures and activities of Trk inhibitor PF-06273340 and General Formula **A** of potential inhibitors used for Trk binding affinity prediction.

The use of a structure-based drug design process enabled the development by Pfizer group of clinical candidate PF-06273340 (Figure 2), a selective pan-Trk inhibitor with a limited brain availability to reduce side effects due to Trk inhibition in the central nervous system. The resolution of the X-ray cocrystal structure of PF-06273340 with TrkA revealed a DFG-out binding mode (PDB ID: 5JFX) [14]. This cocrystal structure was used in further chemoinformatic approaches to predict the binding affinity of a set of Trk inhibitors (General Formula **A**, Figure 2) using enhanced sampling of molecular dynamics with approximation of continuum solvent (ESMACS) and thermodynamic integration with enhanced sampling (TIES). The accuracy of calculated binding affinity of studied TrkA ligands suggested that this computational method could be successfully used in prospective ligand design [15].

**Figure 3:**
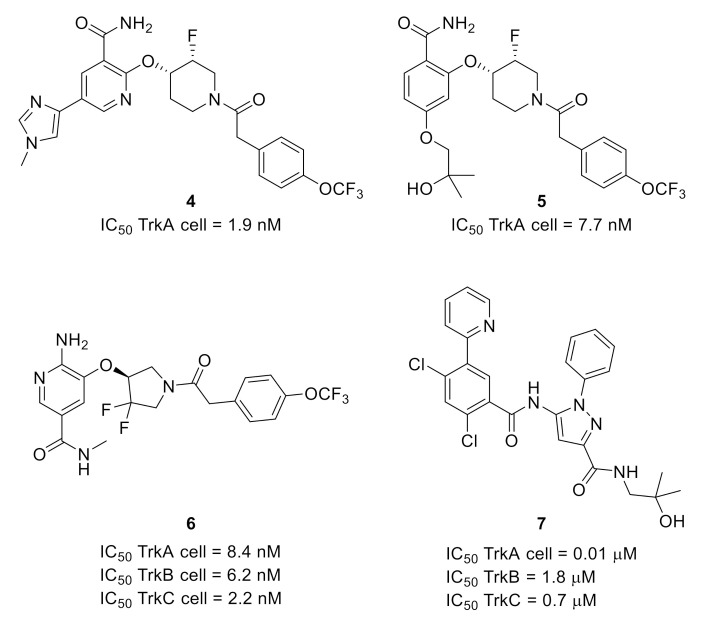
Structures and Trk inhibitory potencies of compounds **4**–**7**.

The same group also developed another series of pan-Trk inhibitors that could avoid the potential clearance prediction liability associated with PF-06273340 metabolization by aldehyde oxidase reaction. Indeed, optimization of a hit identified by screening led to **4**. The structure of **5** is a combination between those of **4** and PF-06273340, while the modification of the linker and the hinge binder gave **6** (Figure 3). These compounds were the most potent synthesized analogs, with nanomolar potencies toward the three isoforms, and showed a better pharmacological profile compared to PF-06273340 [16]. 

Due to the high homology of the ATP-binding site of the three isoforms, most of the ATP-competitive Trk inhibitors are equally active against the three isoforms. By studying an apo TrkA protein crystal structure, researchers from Pfizer noticed that, as previously mentioned, TrkA prefers to adopt a DFG-out autoinhibitory conformation. These structural features were the starting point of a work dedicated to the development of allosteric Trk inhibitors. The most potent compound was pyrazolecarboxamide **7** (Figure 3), targeting an allosteric pocket located behind the ATP-binding site. Compound **7**, exhibiting a selectivity in favor of TrkA versus TrkB and TrkC, is orally bioavailable and demonstrated an interesting profile in preclinical pain models [17].

**Figure 4:**
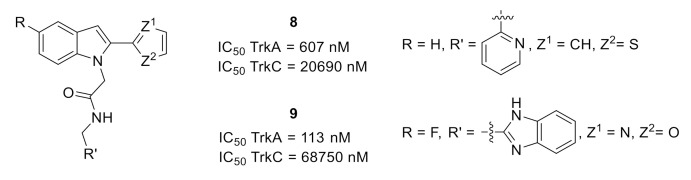
Structures and Trk inhibitory potencies of compounds **8** and **9**.

A design and structure–activity relationship (SAR) process was based on the cocrystal structure of indole **8** with TrkA (PDB ID: 5KMJ). This structure underlined the role of the residues 440–497 from the juxtamembrane region in the selectivity between the three isoforms [18] and led to the synthesis of analog **9** with a better TrkA inhibitory potency and an improved selectivity vs. TrkC (Figure 4) [19].

**Figure 5:**
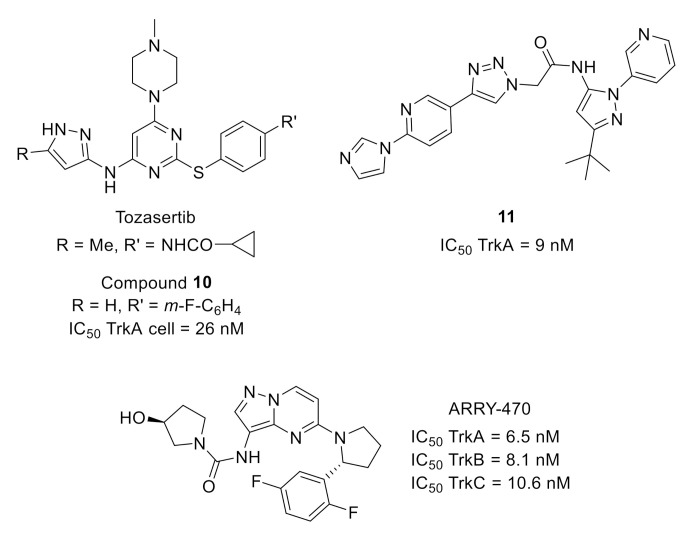
Structures of AurA inhibitor tozacertib and Trk inhibitors **10**, **11** and ARRY-470.

Starting from tozasertib (Figure 5), an Aurora A (AurA) inhibitor developed for cancer application, the use of computer-aided drug design to automate design and selection processes allowed the identification of pyrimidine **10** (Figure 5) with a 10,000-fold improved selectivity toward TrkA versus AurA, nanomolar cellular activity and high selectivity toward a large panel of protein kinases [20].

Optimization of leads identified by high-throughput screening (around 30,000 compounds from kinase or nonkinase programs) led to the identification of potent pan-Trk inhibitors (pyridinetriazole **11**, Figure 5) with in vivo efficacy on inflammatory and neuropathic pain after oral administration. The rational SAR was based on the binding mode of a phenyltriazole analog with TrkA, determined by X-ray crystallography (PBD code: 4PMM), which demonstrated a type II binding mode in this series [21].

Finally, early administration of ARRY-470 (Figure 5), an ATP-competitive selective pan-Trk inhibitor with a nanomolar activity toward TrkA, B and C demonstrated the beneficial effect of Trk inhibition in a mouse model of bone cancer pain [22].

### 2.2. Rearranged During Transfection (RET)

**Figure 6:**
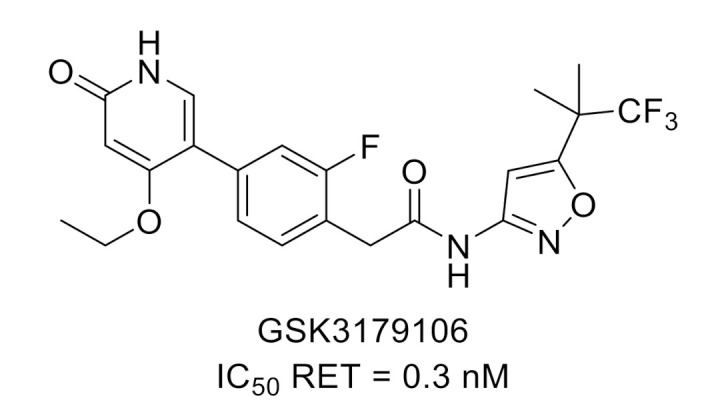
GSK3179106 structure and kinase inhibitory potency toward RET.

Rearranged during transfection (RET) is a transmembrane neuronal growth factor tyrosine kinase receptor activated by binding to a glial cell line-derived neurotrophic factor (GDNF). Numerous papers reported RET inhibitors in cancer application [23,24,25]. However, it has been demonstrated that RET is involved in the development of the enteric nervous system and, therefore, constitutes an attractive target for the discovery of new treatments for visceral pain such as in irritable bowel syndrome. A SAR performed on hits identified by high-throughput screening led to GSK3179106 (Figure 6), a potent and selective RET inhibitor active in vivo in a noninflammatory irritation model of colonic hypersensitivity [26,27].

### 2.3. Epidermal Growth Factor Receptor (EGFR)

**Figure 7:**
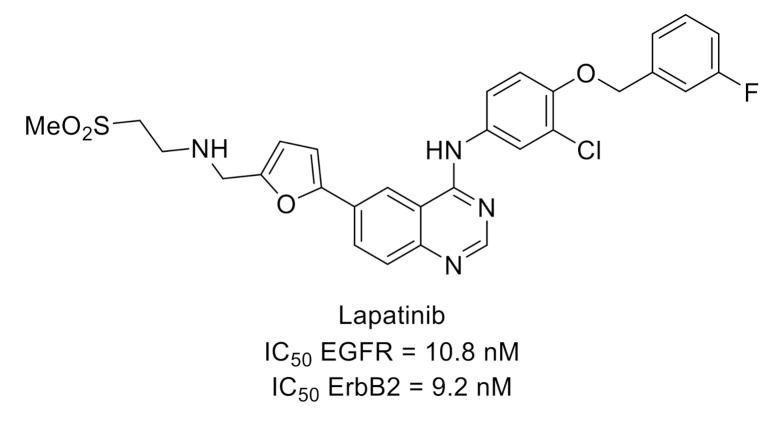
Lapatinib structure and kinase inhibitory potency toward EGFR and ErbB2.

Lapatinib (Figure 7) is a dual inhibitor of EGFR (ErbB1) and ErbB2, members of the epidermal growth factor receptor family, which was approved for treatment of advanced breast cancer. This compound was used on a rat model of whisker pad mechanical allodynia to evidence that orofacial mechanical allodynia was mediated via ErbB3/ErbB2 heterodimers [28,29].

### 2.4. Janus Kinase (JAK)

**Figure 8:**
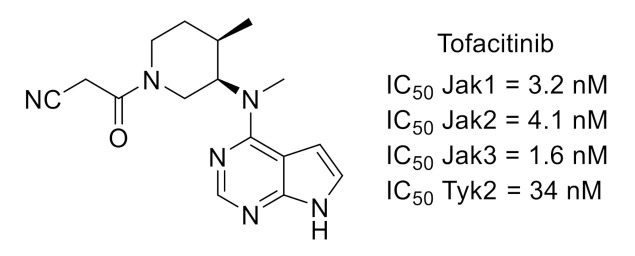
Tofacitinib structure and kinase inhibitory potency toward Jak and Tyk.

The Janus kinase (JAK)–signal transducer and activator of transcription (STAT) pathway is involved in pain modulation [30]. JAKs are nonreceptor tyrosine kinases comprising four members (JAK1, JAK2, JAK3 and tyrosine kinase 2 (Tyk2)). Pfizer group reported several studies showing that CP-690550 (tofacitinib, Figure 8), an orally active, potent and selective JAK inhibitor, alleviated pain in rheumatoid arthritis patients [31,32,33].

### 2.5. Vascular Endothelial Growth Factor Receptor (VEGFR)

**Figure 9:**
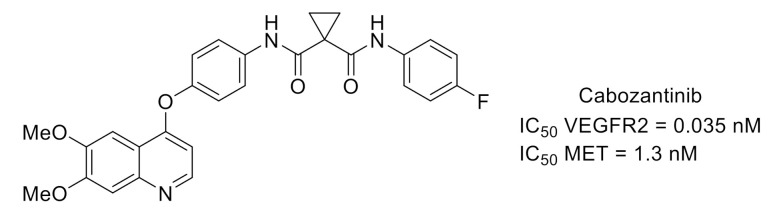
Cabozantinib structure and kinase inhibitory potency toward VEGFR2 and MET.

Cabozantinib (Figure 9) is a potent inhibitor of VEGFR2, also active toward mesenchymal-to-epithelial transition (MET) kinase, used in the treatment of various solid tumors like advanced castration-resistant prostate cancer (CRPC). Both kinases are involved in pathways controlling the development of prostate cancers. These pathologies are frequently associated with bone metastases that are very painful. In a clinical study performed on patients with CRPC, cabozantinib demonstrated clinically relevant pain palliation [34,35].

### 2.6. Src-Family Protein Tyrosine Kinases (SFKs) and Breakpoint Cluster Region (BCR)–Abelson (Abl) Fusion Protein

**Figure 10:**
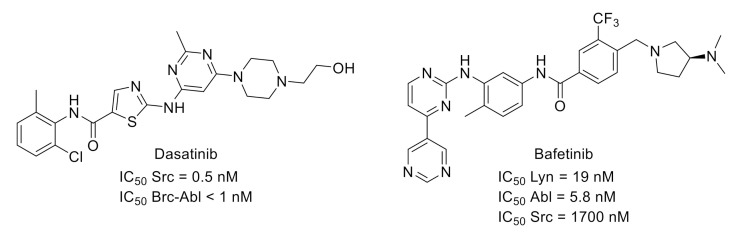
Structures and activities of dual SFK/Brc-Abl inhibitors.

Src-family of protein tyrosine kinases is a group of nonreceptor protein kinases. Two SFK members expressed in the central nervous system (Src and Fyn) are involved in the phosphorylation pathway of *N*-methyl-D-aspartic acid receptor activation, which has an important role in neuronal sensitization in chronic pain. Moreover, intrathecal administration of SFK inhibitors led to alleviated mechanical allodynia in chronic pain models [36]. All these findings indicated that SFKs are interesting targets for pain application. For example, dasatinib (Figure 10) [37], an orally available multitargeted kinase inhibitor with nanomolar potencies toward SFK and Bcr–Abl, demonstrated potential applications in pain symptoms associated with an increased osteoclast activity such as bone metastases [38].

Bafetinib (Figure 10), another multikinase inhibitor targeting Lyn, an SFK family member, and Bcr–Abl, is also of interest for analgesia treatment via its effect on the diminution in PAR2-induced activation of TRPV4 channels and consequent mechanical hyperalgesia [39,40].

### 2.7. FMS-Like Tyrosine Kinase 3 Receptor (FLT3)

**Figure 11:**
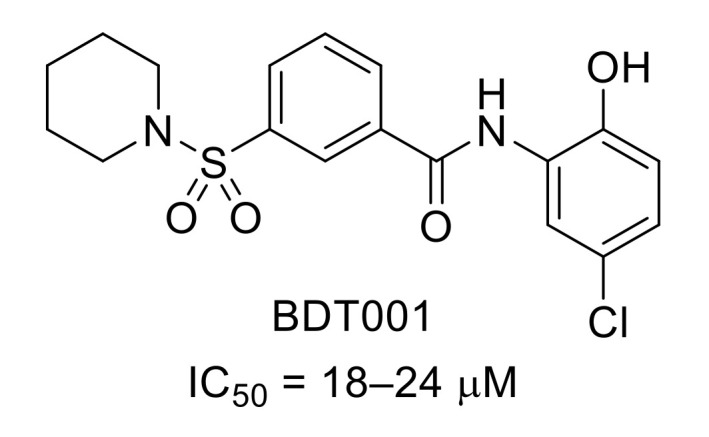
Structure of BDT001 and inhibitory potency of FL-induced FLT3 phosphorylation in RS4-11 cells.

FLT3 tyrosine kinase is expressed in most hematopoietic cells. Recently, it was demonstrated that in dorsal root ganglia, FLT3 is a critical actor in peripheral neuropathic pain initiation and maintenance in mice. Using X-ray crystal structure of FLT3 bound to cytokine FMS-like tyrosine kinase 3 ligand (FL) (PDB ID: 3QS7), as well as in silico screening, hits were identified that effectively prevented extracellular FL binding to FLT3 [41]. This work led to the synthesis and further biological evaluation of BDT001, a noncompetitive FL-binding inhibitor that disrupted the positive cooperativity of FL binding (Figure 11). BDT001 inhibited FL-induced FLT3 phosphorylation in leukemia-derived RS4-11 cells with an IC_50_ of 18–24 µM. It was suggested that BDT001 is an FLT3 negative allosteric modulator. Intraperitoneal administration of BDT001 in mice suppressed FL-induced mechanical hypersensitivity and inhibited mechanical hypersensitivity in the sciatic nerve chronic constriction injury (CCI) model. However, BDT001 did not change CFA-induced inflammatory mechanical pain hypersensitivity.

## 3. CMGC Group

### 3.1. p38 Mitogen-Activated Protein Kinases (p38 MAPKs)

p38 mitogen-activated protein kinases (p38 MAPKs) are a family of serine/threonine kinases widely expressed and consisting of four isoforms: p38α, p38β, p38γ and p38δ. p38 MAPKs are activated by stress signals and inflammatory stimuli, and they contribute to cellular responses associated with neuropathic and inflammatory pain. The function of p38 MAPAKs in the central nervous system and their involvement in pain were recently reviewed [42,43,44]. These protein kinases were early identified as potential targets for pain management and inflammatory diseases. Over the last two decades, numbers of p38 MAPK inhibitors have been reported and evaluated against pain symptoms, including in the course of clinical trials. In addition, some of them have also been used as tools to elucidate their role in signaling pathways. Several reviews have summarized the progress in the field of p38 MAPK inhibitors as analgesic agents [42,43,45,46]. The relevance of p38 MAPK inhibitors for the treatment of pain is overall supported, although the mechanisms involving p38 MAPK in nociception are not fully understood, and no p38 inhibitor has reached the market to date.

**Figure 12:**
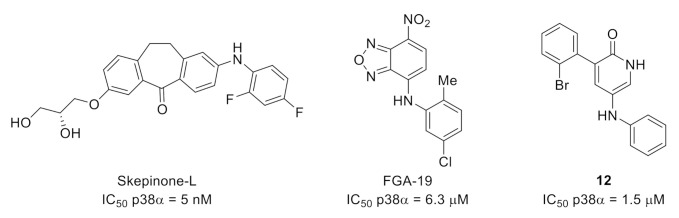
Structures and kinase inhibitory potencies of skepinone-L, FGA-19 and **12**.

Besides drug examples reported in these reviews, new scaffolds evaluated in rodent pain models have emerged. For example, skepinone-L was reported in 2012 as a highly selective and potent p38α/β MAPK inhibitor (Figure 12) [47]. In a study on the role of the spinal p38 in inflammatory and neuropathic pain, intraperitoneal or perineural administration of skepinone-L inhibited mechanical allodynia induced by sciatic nerve CCI in both male and female mice. In contrast, intrathecal injection reduced formalin-induced inflammatory pain and reduced CCI-induced neuropathic pain in male but not female mice or rats, indicating sex-dependent microglia signaling in the spinal cord [48].

FGA-19 is a compound targeting the docking groove of p38 MAPK (Figure 12) [49]. FGA-19 inhibited the phosphorylation of MEF-2A, a docking-dependent substrate, with an IC_50_ value of 6.3 µM and impaired the in vitro interaction between p38 and MK2, another docking-dependent substrate. The mode of action of FGA-19 precludes the inhibition of p38 using a protein substrate lacking a functional docking site. Thus, FGA-19 did not show any p38 inhibitory activity in an in vitro assay using myelin, a protein substrate that appears not to have a functional docking site. FGA-19 was evaluated in vivo in the high-dose carrageenan model of persistent inflammatory hyperalgesia in mice, showing that intrathecal administration of FGA-19 inhibited inflammatory and postinflammatory pain.

Finally, compound **12** is a pyridinone derivative recently reported as a selective p38α MAPK inhibitor, with an IC_50_ value of 1.5 µM (Figure 12) [50]. Its selectivity was demonstrated using a panel of 62 protein kinases including p38 isoforms and protein kinases close to p38 in the phylogenetic tree. Compound **12** was evaluated in vivo for its antalgic properties. It was found to prevent mechanical allodynia in a rat model of CFA-induced inflammatory pain and quickly reverse facial neuropathic mechanical allodynia.

### 3.2. Mitogen-Activated Protein Kinase Kinases (MEKs)/Extracellular Signal-Regulated Kinases (ERKs)

**Figure 13:**
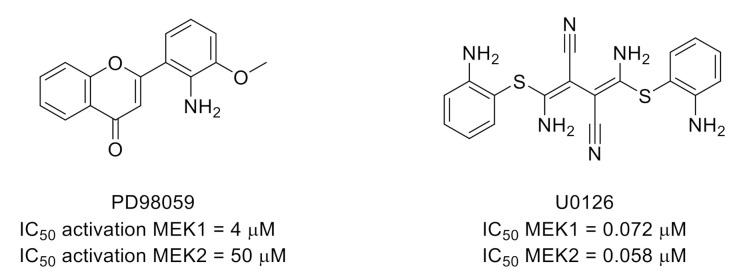
Structures and kinase inhibitory potencies toward MEK1/2 of PD98059 and U0126.

Extracellular signal-regulated kinases (ERKs) 1 and 2 are serine/threonine kinases, also part of the MAPK family. ERK1/2 are involved in many cellular regulation processes and are recognized as important in pain signaling and a target for pain treatment [51]. A recent review described the major roles of ERKs that have been elucidated through pain research [52]. ERK signaling can be altered by inhibition of upstream protein kinases MEK1/2 (Mitogen-activated protein kinase kinase 1/2). Thus, known MEK1/2 inhibitors such as PD98059 [53] and U0126 [54] have been used as tools to study the involvement of this MEK/ERK signaling pathway in pain (Figure 13). For example, U0126 was recently used in a study showing that ERK1/2 phosphorylation contributes to tissue-injury pain hypersensitivity in rats [55].

### 3.3. c-Jun N-Terminal Kinases (JNKs)

**Figure 14:**
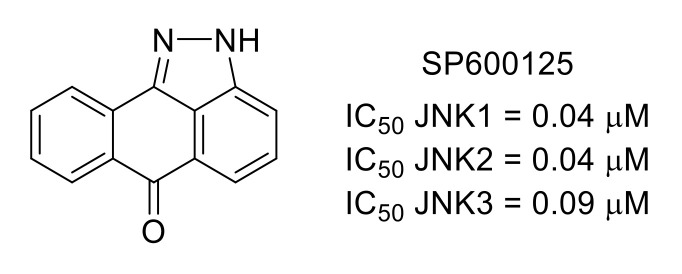
Structure of SP600125 and inhibitory potency toward JNKs.

c-Jun N-terminal kinase is the third major member of the MAPK family, with three isoforms, JNK1, JNK2 and JNK3 [56]. Like the other members of the MAPK family, p38 and ERK1/2, JNKs are involved in the regulation of pain. SP600125 [57] is a commercially available inhibitor of JNK1/2/3 that is frequently used as a molecular tool to study cellular signaling pathways (Figure 14). For instance, SP600125 was recently used in a study showing the role of the JNK pathway in cancer-induced bone pain (CIBP) and decreased mechanical allodynia in CIBP rats [58]. In another example, it was shown that inhibition of JNK using SP600125 prevents pain hypersensitivity induced by antiretroviral zalcitabine and stavudine in mice [59].

### 3.4. Apoptosis Signal-Regulating Kinase 1 (ASK1)

**Figure 15:**
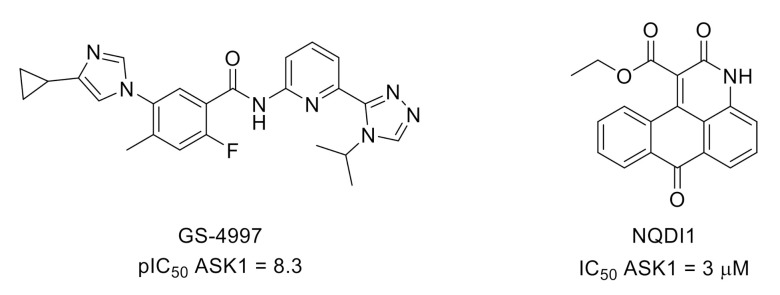
Structures and ASK1 inhibitory potencies of GS-4997 and NQDI1.

Apoptosis signal-regulating kinase 1 (ASK1) is a mitogen-activated protein kinase kinase kinase (MAPKKK) and, therefore, an upstream member of the MAPK signaling pathway that regulates p38 and JNK. As a converging point of cell stress signaling, inhibition of ASK1 is of interest for many human diseases, including chronic pain management [60,61]. In recent examples, ASK1 inhibitors GS-4997 (selonsertib) [62] and NQDI1 [63] attenuated mechanical allodynia and thermal hyperalgesia induced by CCI of the sciatic nerve in rats [64,65] (Figure 15). 

### 3.5. Cyclin-Dependent Kinases (CDKs)

**Figure 16:**
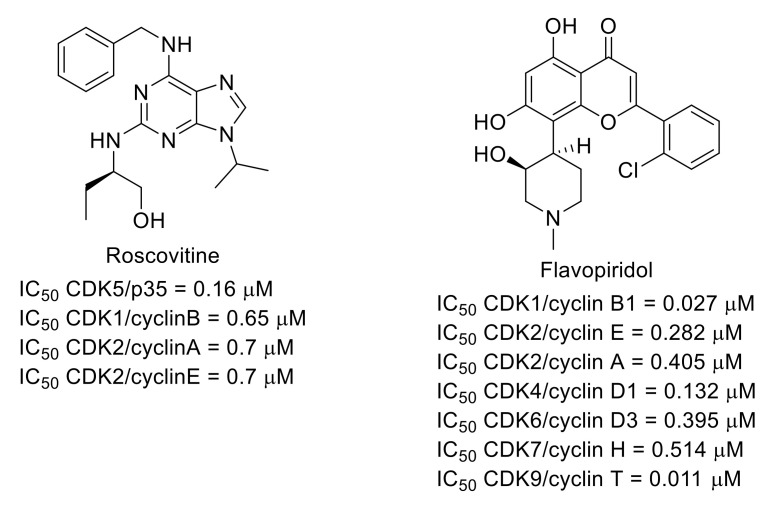
Structures of roscovitine and flavopiridol and inhibitory potencies toward CDKs.

Among the cyclin-dependent kinases (CDKs), the role of CDK5 in pain signaling was identified in the 2000s [66,67,68,69]. Therefore, some CDK inhibitors were used to explore the function of this family of protein kinases in pain. For example, it was shown that CDK5 inhibitor roscovitine [70] (Figure 16) has an analgesic effect in animal pain models [71]. Nonselective CDK inhibitor flavopiridol (for CDK inhibitory activities, see [72]) (Figure 16) also limited spinal cord injury-induced spontaneous pain and the development and maintenance of hyperesthesia [73].

### 3.6. CDC-Like Kinase 2 (CLK2)/Dual-Specificity Tyrosine Phosphorylation-Regulated Kinase 1A (DYRK1A)

**Figure 17:**
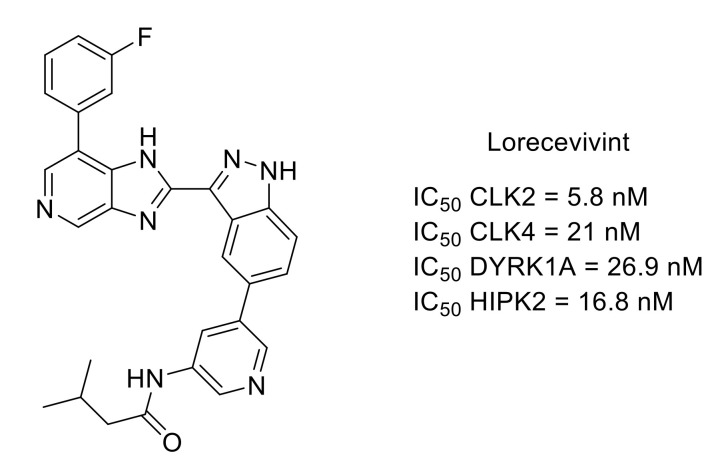
Structure and kinase inhibitory potencies (IC_50_ values < 30 nM) of lorecivivint.

Lorecivivint is a protein kinase inhibitor that demonstrated modulation of the Wnt pathway, chondrogenesis, cartilage protection and anti-inflammatory properties (Figure 17). The primary target of lorecivivint is CDC-like kinase 2 (CLK2) with an IC_50_ value of 5.8 nM. Other protein kinases are also potently inhibited in vitro within the CMGC group such as CLK4, dual-specificity tyrosine phosphorylation-regulated kinase 1A (DYRK1A) or homeodomain-interacting protein kinase 2 (HIPK2). However, it was shown that CLK2 and DYRK1A were important for the activity of lorecivivint [74]. Lorecivivint is currently undergoing Phase III clinical evaluation in subjects with knee osteoarthritis. Results of Phase II showed the potential of lorecivivint for osteoarthritis and associated analgesia [75,76].

### 3.7. Glycogen Synthase Kinase 3 (GSK3)

**Figure 18:**
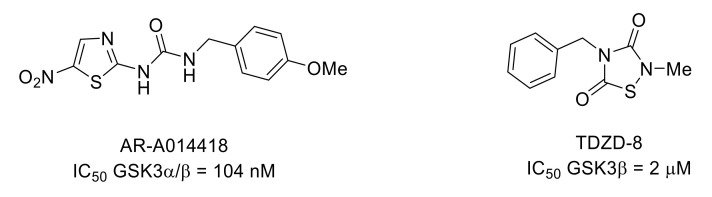
Structure and GSK3 inhibitory potencies of AR-A014418 and TDZD-8.

Glycogen synthase kinase 3 (GSK3) is a serine/threonine kinase that occurs in two isozymes in mammals: GSK3α and GSK3β. In particular, GSK3β plays a role in neuroinflammation and pain [77,78]. AR-A014418 is an ATP-competitive inhibitor of GSK3 with an IC_50_ value of 104 nM (Figure 18). This compound produced an analgesic effect in several mouse models of nociception [79,80]. It was also shown that GSK3 inhibitor TDZD-8 (Figure 18) attenuated remifentanil-induced mechanical and thermal hyperalgesia in rats [81].

### 3.8. Casein Kinase 2 (CK2)

**Figure 19:**
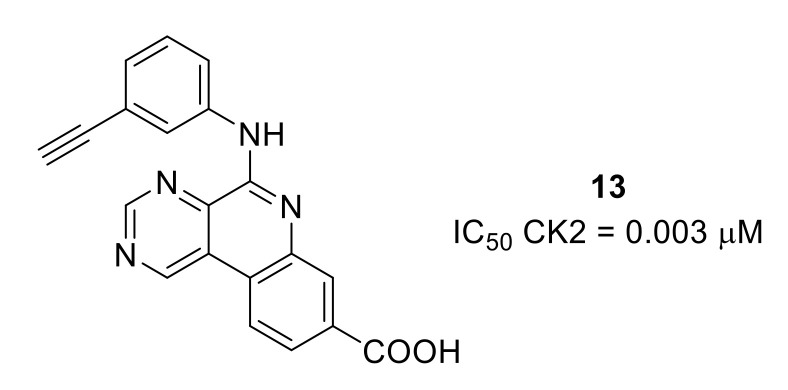
Structure and CK2 inhibitory potency of compound **13**.

Serine/threonine kinase CK2 has been widely targeted for cancer applications. However, it was reported that CK2 can be involved in the modulation of pain [82]. A rationally designed SAR study starting from a selective ATP-competitive CK2 inhibitor developed for cancer application led to the discovery of compound **13**, an orally available nanomolar CK2 inhibitor that reduced formalin-induced pain in mice (Figure 19) [83].

## 4. Protein Kinases A, G and C (AGC) Group

This group consists of more than 60 serine/threonine kinase members involved in numerous signaling pathways, including metabolism, cell proliferation and pain. 

### 4.1. PKA, PKB, PKC, PKMζ and PKG

**Figure 20:**
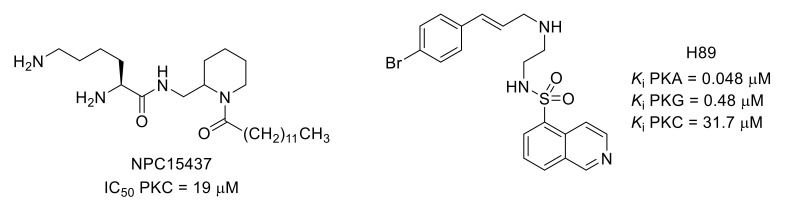
Structures and kinase inhibitory potencies of NPC15437 and H89.

PKA and PKC are involved in hyperalgesia by phosphorylating excitatory neurotransmitter receptor AMPAR GluR1 subunit. For example, NPC15437, a competitive selective PKC inhibitor, and H89, a PKA inhibitor, alleviated hyperalgesia in a remifentanil-induced model (Figure 20) [84,85,86,87]. Among multiple PKC isoforms, PKCγ and PKCε have been reported as key mediators of pain [88]. The problem of isoform selectivity for small molecule protein kinase inhibitors has been overcome by the use of peptides that compete with activated PKC for binding to the isoenzyme-specific docking proteins, receptors for activated C kinase [89]. For example, peptide KIG31-1, known as a selective PKCγ inhibitor, was used to evidence the contribution of PKCγ activation to the manifestation of phorbol 12,13-dibutyrate (PDBu)-induced mechanical allodynia [90].

PKMζ is an *N*-terminally truncated constitutively active form of the atypical PKC that is also involved in the regulation of GluR1-AMPA receptor trafficking [91]. ζ-Pseudosubstrate inhibitory peptide (ZIP) was used to reveal the role of spinal PKMζ and demonstrated that this kinase could be of interest to prevent surgery-induced chronic pain in patients [92,93]. The use of ZIP in an animal neuropathic pain model also suggested that PKMζ could be involved in the modulation of neuropathic pain via motor cortex stimulation [94].

**Figure 21:**
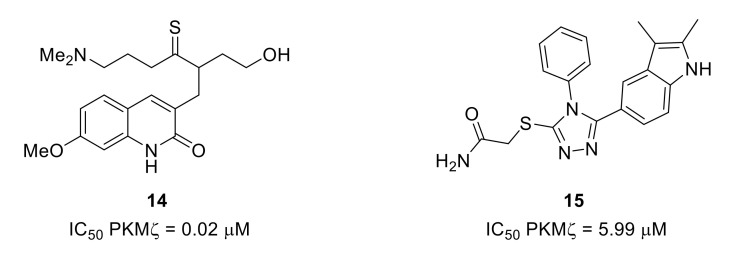
Structures and inhibitory potencies of PDMζ inhibitors **14** and **15**.

The design of ATP-competitive PKMζ inhibitors based on a model built by sequence homology from PKCι led to quinolinone **14** and triazole **15** (Figure 21). Due to cytotoxicity problems, quinolinone **14**, the most potent PKMζ inhibitor of the series, was not used in cellular assays. However, the triazole derivative with lower PKMζ inhibitory potency led to reduced expression of proinflammatory mediators in cells [95].

**Figure 22:**
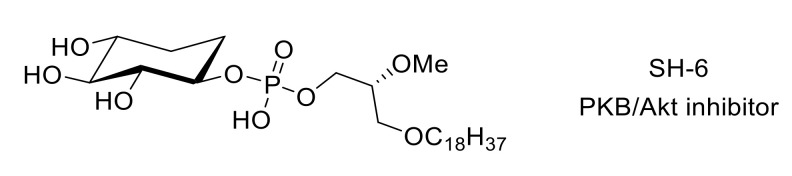
Structure of compound SH-6.

The use of inhibitors such as SH-6 [96] (Figure 22) demonstrated the involvement of PKB/Akt in mechanical hypersensitivity [97]. More recently, Cheng et al. studied the role of phosphatidylinositol 3-kinase (PI3K)/PKB/mTOR signaling pathway in neuropathic pain caused by nucleoside reverse transcriptase inhibitors in HIV treatment [98].

**Figure 23:**
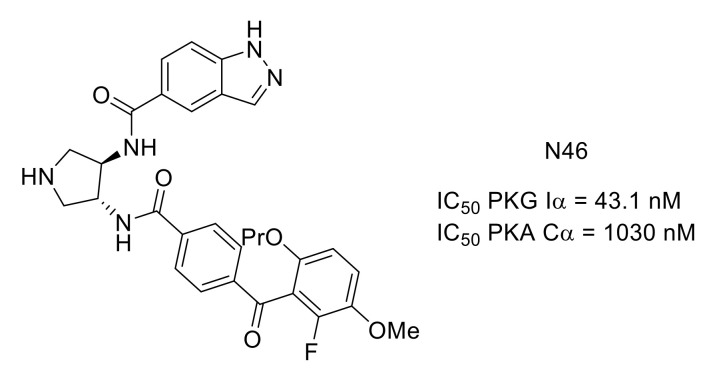
Structure and kinase inhibitory potency of N46.

Activation of PKG Iα is involved in long-term hyperexcitability leading to chronic pain. PKG Iα C-domain and PKA Cα are highly homologous with 45% sequence identity. However, N46 is a selective PKG Iα nanomolar inhibitor exhibiting good selectivity over PKA (Figure 23). Comparing the crystal structure of N46 bound to PKG Iα C-domain (PDB ID: 6C0T) and PKA Cα (PDB ID: 6C0U), the selectivity of N46 in favor of PKG was explained by steric hindrance [99]. This compound was able to reduce thermal hyperalgesia and pain osteoarthritis in animal models [100]. 

### 4.2. Rho-Associated Coiled-Coil-Containing Kinase (ROCK)

**Figure 24:**
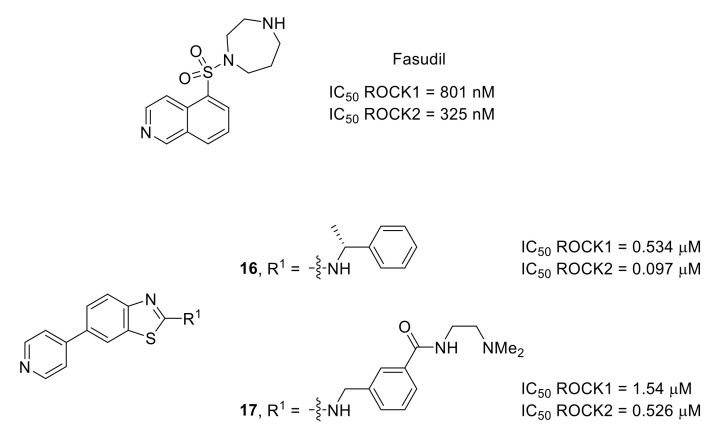
Structures of fasudil and compounds **16** and **17**. IC_50_ values determined in the presence of 100 μM ATP concentration.

ROCKs, involved in Rho GTPase pathway, are promising targets for neurological disorders [101]. This kinase family contains two highly homologous isoforms: ROCK1 and ROCK2. Fasudil (Figure 24), the first marketed ROCK1/2 inhibitor containing an isoquinoline moiety, was used in various preclinical models of pain. The results obtained demonstrated that ROCKs are involved in different pain states from osteoarthritic to neuropathic pain and possibly in spontaneous pain [102]. A designed SAR study allowed the identification of new ROCK1/2 inhibitors based on a 2-aminobenzothiazole scaffold. Both compounds **16** and **17** demonstrated interesting activities in vivo in a mechanical hyperalgesia model (Figure 24) [103].

**Figure 25:**
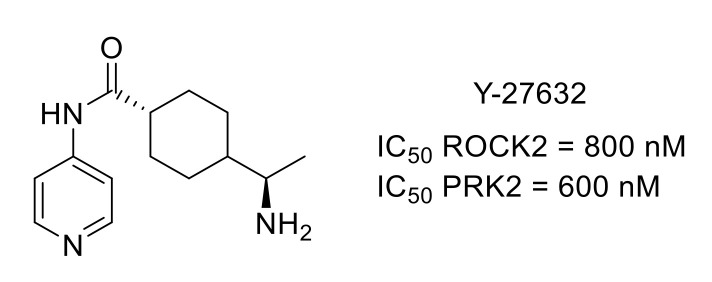
Y-27632 structure and kinase inhibitory potency.

Y-27632 is an ATP-competitive ROCK inhibitor, also active toward protein kinase N2 (PKN2), a PKC-related protein kinase [104]. Y-27632 was used to demonstrate the central role of these protein kinases in neuropathic pain, highlighting the potential of protein kinase inhibitors to treat neuropathic pain (Figure 25) [105].

## 5. Ca^2+^/Calmodulin-Dependent Protein Kinase (CAMK) Group

**Figure 26:**
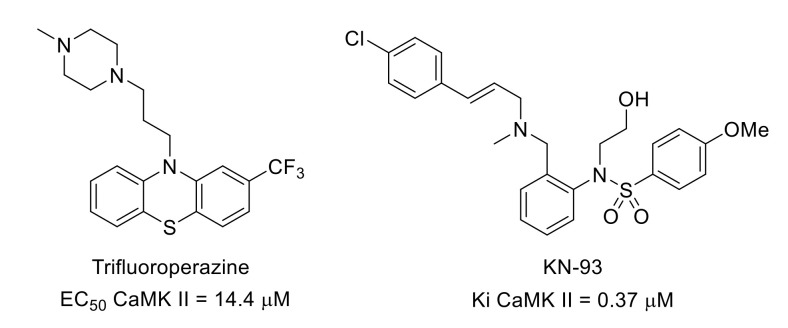
Structures and CAMK II inhibitory potency of trifluoroperazine and KN-93.

CaMK II is a serine/threonine kinase, part of the CAMK group, that is activated by the Ca^2+^/calmodulin complex. There are different CAMK II isoforms such as CaMK IIα and CaMK IIβ. The intracellular level of Ca^2+^ is significantly increased in chronic pain. It has been shown that inhibiting the CaMK II signaling pathway could be a strategy to attenuate neuropathic pain. For example, pain reduction was observed in sickle cell disease patients treated with trifluoroperazine (Figure 26), a CaMK II inhibitor [106].

Trifluoroperazine and KN-93 (Figure 26), another calmodulin-competitive CaMK II inhibitor [107], also reversed neuropathic/inflammatory pain in mice [108,109]. It was also demonstrated that by inhibiting spinal CaMK II phosphorylation, KN-93 reduced postoperative hyperalgesia in rats [110].

## 6. Casein Kinase 1 (CK) Group

**Figure 27:**
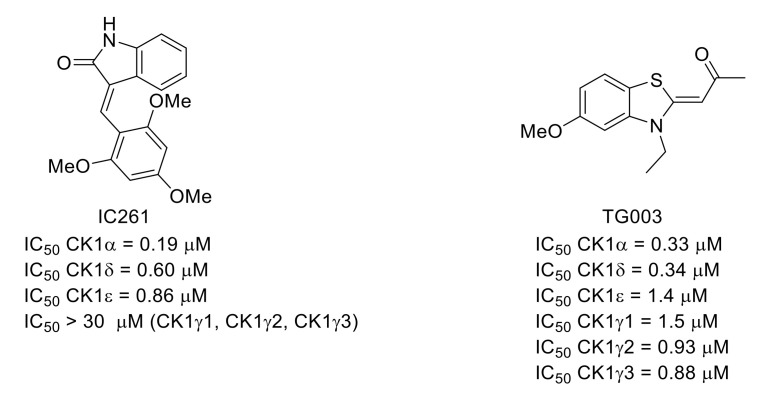
Structures and CK1 inhibitory potencies of IC261 and TG003.

Serine/threonine casein kinase 1 family (CK1) is composed of various highly homologous isoforms. IC261 (Figure 27) is a conformation-selective ATP-competitive CK1 inhibitor identified by high-throughput screening [111]. In a same study, the effects of IC261 and TG003 (a potent CLK inhibitor: IC_50_ CLK1 = 20 nM, IC_50_ CLK2 = 200 nM, IC_50_ CLK3 > 10 µM, IC_50_ CLK4 = 15 nM [112]) were studied in vivo on carrageenan- or CFA-induced models of inflammatory pain. Both compounds decreased mechanical allodynia and thermal hyperalgesia [113].

## 7. Atypical and Other Protein Kinase Groups

### 7.1. IκB Kinases (IKKs)

**Figure 28:**
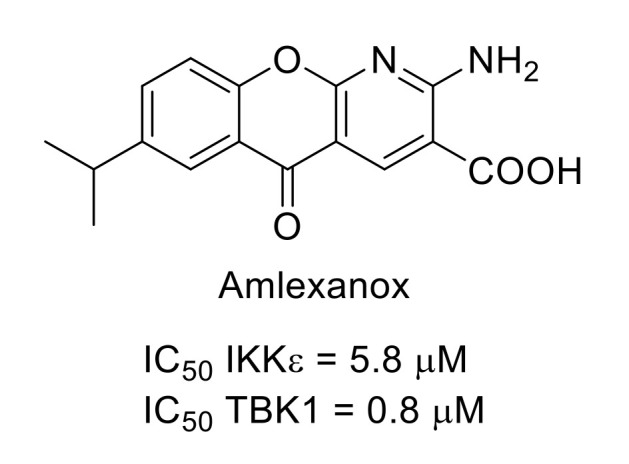
Amlexanox structure and kinase inhibitory potency.

This serine/threonine protein kinase group contains four members: IKKα, IKKβ, IKKε and TANK-binding kinase 1 (TBK1). IKKε knock-out attenuates pain-like behavior in the neuropathic pain model. The use of amlexanox (Figure 28), an ATP-competitive IKKε/TBK1 inhibitor [114,115], also led to reduced mechanical hyperalgesia and cold allodynia, showing that IKKs are relevant targets for neuropathic pain treatment [116].

### 7.2. Mammalian Target of Rapamycin (mTOR)

This atypical serine/threonine kinase, involved in the phosphoinositide 3-kinase (PI3K)/Akt pathway and participating in two protein complexes, mTOR complex 1 (mTORC1) and mTOR complex 2 (mTORC2), is a well-known target for cancer therapy.

**Figure 29:**
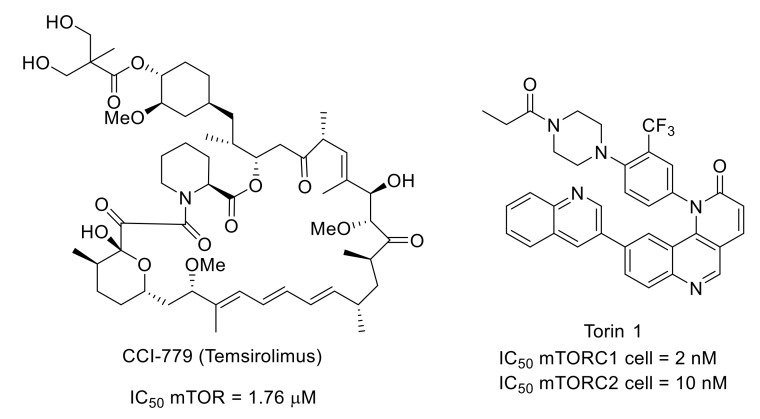
Structures and mTOR inhibitory activity of CCI-779 and Torin 1.

However, numerous works also described mTOR signaling pathways as key targets for novel chronic pain therapies. Indeed, systemic injection of CCI-779 (temsirolimus), a rapamycin analog with a better aqueous solubility, led to a decrease in mechanical and cold hypersensitivity in mouse neuropathic and inflammatory pain, highlighting the implication of mTORC1 in the pain signaling pathway. The same results were obtained with Torin 1, an mTORC1/smTORC2-selective ATP-competitive inhibitor identified by rationally directed library synthesis (Figure 29) [117,118,119,120].

## 8. Conclusions

The human kinome consists of more than 500 protein kinases that share the same natural substrate (ATP). Most of the known inhibitors target the kinase ATP-binding site with a heteroaromatic moiety able to mimic the ATP adenine moiety. This ATP-competitive inhibition type frequently leads to cross-inhibitions. However, the development of protein kinase inhibitors was successful for cancer therapy, with more than 40 currently marketed drugs, mainly targeting tyrosine kinases. Other protein kinase inhibitors binding to different kinase domains, such as allosteric inhibitors, have been designed to achieve better selectivity in protein kinase inhibition profiles. Besides cancer therapy, these ubiquitous biological targets are also emerging as targets of interest for other pathologies such as pain symptoms. As highlighted by the work reported in this review, various protein kinases belonging to different kinome groups are now studied for their role in pain as targets for protein kinase inhibitors. Whether used as tools to study signaling pathways or as drug candidates, protein kinase inhibitors have demonstrated interesting activities in various animal pain models, providing support for further development of pain management options. However, we can observe that among the large number of protein kinase inhibitors reported in the literature, few are evaluated for their analgesic properties when targeted protein kinases are relevant in the context of pain management. One reason could be that research projects dedicated to kinase inhibitors in pain management are underrepresented, despite offering opportunities for the development of novel pain treatments. A second reason is probably linked to the access to facilities for evaluation of novel drugs against pain, with costly in vivo testing on animal models, beyond widespread in vitro protein kinase inhibition assays. Therefore, the development of novel protein kinase inhibitor drug candidates for the treatment of pain is challenging and still requires a better understanding of the role of protein kinases in pain and of mechanisms underlying pain symptoms. Intense efforts toward drug discovery leading to the development of specific inhibitors and further biological evaluation of their analgesic properties should be encouraged and would favor the discovery of new therapeutic approaches, including protein kinase inhibition, for better pain management.

## Data Availability

Not applicable.
